# Evolving optimal text clusters: A novel GA-driven framework for dynamic ensemble fusion of multi-model contextual embeddings

**DOI:** 10.1371/journal.pone.0353506

**Published:** 2026-07-13

**Authors:** Ali Sabah, Zaid Alaa

**Affiliations:** Department of Computer Science, Faculty of Education for women, University of Kufa, Najaf, Iraq; University of Kerbala, IRAQ

## Abstract

Text clustering is an essential activity in unsupervised natural language processing (NLP), and allows the automatic structure of large-scale textual collections (in natural language processing) in news classification, routing of technical questions, and text summarisation. With the emergence of contextual embedding models, such as SBERT, RoBERTa, and DistilBERT, there has been a significant improvement in the quality of clustering, with these models producing dynamic and context-sensitive representations that are more likely to reflect domain-specific semantics than the traditional word embeddings of Word2Vec and GloVe. Although the models of contextual embedding have their own advantages, they show varying performance in different domains and the current ensemble techniques propose fixed, flat fusion weights which do not utilize their complementary abilities. There is no current solution to dynamic, label-free optimisation of weight to multi-model contextual embedding fusion in unsupervised clustering which would bridge a major gap between fixed integrative approaches and adaptive, domain-accommodative model combination. This paper presents a new Genetic Algorithm (GA)-based ensemble model that dynamically optimizes the best fusion weights of SBERT, RoBERTa, and DistilBERT embeddings without ground-truth labels. The hypothesis is that evolutionary optimisation can discover domain-adaptive weight sets that outperform standalone models as well as fixed ensemble baselines on linguistically heterogeneous datasets. The framework uses L2-normalisation and topology in which the topology is a sum-to-one constraint so that the models can be fairly integrated, and a composite fitness measure based on Silhouette Score, Adjusted Rand Index, and Topic Coherence to direct the weight evolution through tournament selection, uniform crossover, and Gaussian mutation. The proposed framework on three heterogeneous benchmarks, AG News, 20 Newsgroups, and Stack Overflow, has Silhouette Score improvements of +14–16 percent, and Topic Coherence gains of +17 percent, all statistically significant at p < 0.05. Evolved weights can be completely understood, depicting domain-specific model roles, which guide future architecture choice. The framework is suggested as a scalable, modular system to practical unsupervised NLP problems, and its architecture can be easily extended to new transformer systems and semi-supervised systems.

## 1. Introduction

Text clustering is an improvement method in unsupervised natural language processing (NLP) and plays an essential role in arranging unstructured textual data in many arenas, and discovery of hidden patterns in uncategorized data [1,2]. Traditional approaches trust on static word representations like GloVe [3] or Word2Vec [4] that provide a fixed set of vectors to words and do not take context into account. However, the techniques are not easy to identify lexical ambiguity (e.g., a riverbank and a financial institution) and complicated semantic relations foremost to poor clustering results [5]. The occurrence of contextual embeddings in the model of BERT [6], RoBERTa [7], and their modifications has transformed NLP as it produces dynamic, context-sensitive representations that alteration to adapt with the surrounding text. Such models increase the linguistic nuances encoding with high clustering accuracy of intricate datasets [8]. Contextual embeddings (e.g., SBERT, DistilBERT, and RoBERTa) are different to fixed representations (GloVe, Word2Vec) as they produce dynamic and context-sensitive vectors that identify domain-specified semantics (e.g., technical jargon in Stack Overflow or similar topics in AG News). Despite the fact that contextual embeddings are superior to the static models, all of their performance vary in domains and tasks. To illustrate, RoBERTa is shown to be extremely effective in finding technical jargon in Questions in Stack Overflow [9], whereas DistilBERT is shown to be efficient in large-scale data [10]. This variability initiates the desire to use ensemble techniques where predictions of many models are used to mitigate bias and inconsistency [11]. But the averaging of the contribution of different models in a simplistic way often makes it hard to make the most of their corresponding capacities. To address this gap, this paper proposed the integration of Genetic Algorithms (GAs) [12] which is a group of evolutionary optimization methods, to dynamically optimize weights of contribution of each model. The GAs clearly overcome clustering challenges by exploiting the population-based exploration and diversity (through mutation), which otherwise cannot be achieved in traditional algorithms like a grid search or Bayesian optimization in the high-dimensional embedding spaces. Unlike extensive hyperparameter searches (grid search), or enclosed probabilistic searches (Bayesian optimization), GAs rely on tournament selection, cross-over, and Gaussian mutation to avoid local optima and evolve domain-specific fusion schemes. GAs enable adaptive combination of embeddings by refining weights with an iterative process of refinements using quality metrics of clustering (e.g., silhouette score or Adjusted Rand Index [ARI]) [13]. This GA-driven optimization, and ensemble learning have high clustering performance, which is domain-independent. To substantiate the thought of whether the framework could be replicated in the linguistic diversity, technicality and scale, the present study chose datasets (i.e., AG News, 20 Newsgroups and Stack Overflow). AG News investigates label placement of short headlines; 20 Newsgroups judges the strength of casual text where there is a topic overlap and Stack Overflow judges scalability of technical domains that need contextual accuracy. Because of these inadequacies, this paper has the following contributions towards science:

- Novel ensemble-based GA-driven unsupervised contextual embedding fusion. It suggests a novel framework that optimizes Genetic Algorithms to evolve fusion weights of heterogeneous contextual embedding models SBERT, RoBERTa and DistilBERT without ground-truth labels. Although previous GA algorithms in NLP are applied to supervised tasks and earlier evolutionary clusterization algorithms utilize single-model contextual embeddings, the suggested framework is the first to include multi-model contextual embeddings and apply GA-based weight evolution under an unsupervised goal, with L2-normalisation and a sum-to-one constraint to ensure fair and interpretable model integration.
- Simultaneous geometric and semantic optimization using dataset adaptive composite fitness. A novel fitness measure is constructed, which simultaneously maximizes the geometric cluster separation, label fidelity, and intra-cluster semantic coherence on one evolutionary signal. In contrast to earlier algorithms which made use of entirely geometric metrics, the function learns metric weighting on a per-dataset basis, allowing the framework to be used on both labeled and completely unlabeled corpora a property that has not been made possible by existing ensemble clustering methods.
- Cross-domain generalization and systematic benchmark of standalone contextual models. Standalone SBERT, RoBERTa and DistilBERT are rigorously empirically benchmarked on three linguistically different datasets AGNews, 20 Newsgroups and StackOverflow to quantify the performance limits of individual architectures and offer a community-reusable baseline. Cross-domain assessment then shows the versatility of the framework to formal, informal and technical text, and developed weights indicate domain-specific model roles that cannot be revealed by black-box ensemble methods.

This can be accomplished by reducing the distance between evolutionary computation and deep contextual learning so that this project can further extend the state-of-the-art text clustering to offer scalable and human-understandable solutions to real NLP problems. Integrating GAs and contextual embedding can be regarded as a paradigm shift in unsupervised learning. In the latter, evolutionary optimization allows for dynamic harmony in the capacities of diverse Transformer-based models (e.g., SBERT, RoBERTa, and DistilBERT). This hybrid approach surpasses conventional clustering pipelines by resolving two essential restraints of modern NLP: (1) the inadequate combination of heterogeneous embedding architectures and (2) the absence of flexible approaches for domain-specific text investigation. Distinct from static ensemble methods employing fixed weighting systems, the current GA-driven framework transforms model contributions following dataset-specific features, such as technical jargon in Stack Overflow, linguistic vagueness in 20 Newsgroups, or brief wording in AG News headlines.

## 2. Related studies

NLP has been transformed in contextual embeddings through substitution of static word depictions (e.g., Word2Vec [3], GloVe [4]) with dynamic, context-aware versions. This research comprehensively explain three basic models and their distinct contributions to clustering tasks as follows:

SBERT (Sentence-BERT) [14,15] SBERT adds to BERT [10,16] by presenting a Siamese or triplet network architecture to produce fixed-length sentence embeddings augmented for semantic resemblance. Unique from standard BERT, which yields token-level embeddings which necessitate manual pooling (e.g., mean or [CLS] token), SBERT adds a pooling layer (mean, max, or attention-weighted) in the pretraining for a direct production of sentence vectors. This model undergoes training on Natural Language Inference (NLI) datasets (e.g., SNLI, MultiNLI) using contrastive deficit, which takes full advantage of cosine resemblance between semantically analogous sentences while separating disparate pairs. Its construction removes repetitive BERT calculations for sentence pairs, markedly enhancing inference efficacy. In clustering tasks, SBERT highly performs owing to its uniform vector space, which contains spatially proximate texts that are semantically analogous. To illustrate, in Stack Overflow, SBERT can categorize questions on “React hooks” versus “Angular directives” by identifying complex semantic variances, rendering it exceptional for applications that need fast and interpretable clustering.

RoBERTa (Robustly Optimized BERT Approach) [7] RoBERTa is a further development of BERT, which optimizes training algorithms and hyperparameters. It substitutes BERT’s static masking with dynamically created masked tokens while training, boosting generalization by revealing the model to several contextual variations. RoBERTa also uses longer epochs (up to 500 k steps) and larger batch sizes (8,000 vs. 256 in BERT) and thus improves the convergence and stability. One notable structural change is the removal of the Next Sentence Prediction (NSP) task of BERT, replaced by segment-pair + −inference to downstream tasks. These transformations give RoBERT a deep contextual insight, making it outstanding in areas with technical or unclear language. In AG News, RoBERTa distinguishes between similar categories, like the world and business, by modeling abstruse discourse patterns. Its strength to polysemy (e.g., “Apple” as a company versus a fruit) guarantees uncluttered cluster boundaries, including datasets with high lexical vagueness.

DistilBERT [10,16] is a lightweight variant of BERT that has been kept in its purified form and retains 97% of the performance of BERT with minimal computational cost. It gets to this state through refining the knowledge by training a smaller student model (66 M parameters vs. BERT-base 110 M parameters) to behave like BERT through logit distillation (corresponding yield prospects) and attention distillation (positioning attention head weights). The result of this process is a 60x faster inference rate with an insignificant loss in precision, which makes DistilBERT extraordinary in large-scale clustering workloads like in queries to Stack Overflow (millions of queries). Despite the fact that the DistilBERT one is slightly less complex than the RoBERTa, its effectiveness enables real-time pipelines clustering in low-resource environments. Its trade-off between speed and veracity makes it particularly credible when paired with GA-driven ensemble models, which compensates low precision drops by a dynamic compensatory contribution of more high-fidelity models like RoBERTa.

Some methods that have been used to cluster texts using contextual embeddings are KMeans [17,18], DBSCAN [19], and hierarchical clustering. Nonetheless, such models are mostly treated as black-box feature extractors in research, in spite of the potential to dynamically combine their capabilities. An example would be that SBERT is preferred in semantic similarity-based clustering (such as collecting related news articles) whereas RoBERTa is better at domain-specific clustering (such as technical Stack Overflow threads). DistilBERT is comparable to large-scale clustering that basically demands speed (e.g., analysis of social media in real-time). Standalone models, although individually capable, have the challenge of adapting to problem-specifics of the data, including balancing efficacy and veracity or disentangling overlapping topics. In this regard, the study also advances previous techniques by proposing a GA-based ensemble that allows dynamically balancing the capacities of these models without constraints that limit them individually through evolutionary optimization.

Ensemble learning has emerged as a paradigm of authority in natural language processing (NLP), combining predictions of many models to enhance strength, generalizability and performance on a variety of tasks. Utilizing the respective capabilities of individual models enables ensemble techniques to reduce the danger of overfitting, decrease variance, and improve veracity. The three most prevalent strategies characterize this field: stacking, voting and hybrid architectures, and each has its own rewards and concerns of NLP application. Stacking is based on dynamic weight predictions of base models (e.g., BERT and RoBERTa) using a meta-model (e.g., logistic regression) as an input to their output [20]. The hierarchical model is effective in supervised tasks like classification, where labeled training data guide the meta-model to prefer high-performing models (e.g., use BERT on syntax, RoBERTa on semantics). Its reliance on ground-truth labels and computational complexity however limit its use in unsupervised clustering that does not have labels and critically needs to be calibrated dynamically [20].

Voting sums make predictions through majority voting (hard voting) or probability averaging (soft voting) [21]. Hard voting selects the most frequent forecast of the class, and soft voting selects the most average forecast of the class. Although its sorting method is simple and effective, its results depend on the existence of multiple models (homogeneous models, such as multiple variants of BERT) which regularly produce related errors. There is also a problem of adaptation to domain-specific issues in static systems (e.g., technical language vs. colloquial language), and therefore the lack of flexibility in dynamic clustering configurations [21,22].

Hybrid models join constructions (e.g., Transformers + CNNs) to take advantage of corresponding capacities, such as global attention (Transformers) and local pattern detection (CNNs) [23]. These systems enhance activities like machine translation but are faced by the issue of clustering that are brought about by skewed embedding spaces and training tasks. An unlabeled data that continues to make it challenging to optimize mismatched architectural objectives (e.g., the semantics of SBERT and the efficiency of DistilBERT), limiting their use in unsupervised contexts despite their hypothetical potential [23].

Despite their success in the supervised NLP tasks, the ensemble learning techniques are faced by two significant challenges in the unsupervised clustering namely that of two critical limitations. To begin with, they are limited by supervision bias: algorithms like stacking and voting require labeled data to normalize the weights of models or to support predictions [20,21]; but clustering problems do not have ground-truth labels, forcing them to rely on random or fixed weighting schemes. Second, fixed weighting impedes flexibility – existing ensembles repeatedly apply fixed, existing weights (e.g., equivalent shares of SBERT, RoBERTa, and DistilBERT) [11], without addressing dataset-specific issues such as technical jargon in Stack Overflow (where RoBERTa contextual complexity shows excellent results) or scale requirements in AG News (where DistilBERT efficacy is central). This dogmatism threatens inadequate clustering by its insensitivity to the tradeoffs between veracity, computational cost, and domain-specific language patterns. Such vulnerabilities highlight the urgency of using adaptive ensemble models that dynamically adapt model contributions when no labeled data is available- an issue directly addressed by the GA-driven methodology proposed in this study.

Inspired by the concepts of natural selection and genetic variation, GAs are population-based metaheuristics that give sound responses to complex optimization issues in machine learning (ML). GAs uses process simulation including, selection, crossover, and mutation, to explore iterative evolution of potential results (e.g., feature subsets, hyperparameters, or model weights) to maximize a given fitness function. Their adaptability has led to wide usage in the three central areas of ML listed below: Feature Selection: GAs are effective in identifying the best feature subsets of high-dimensional data to reduce computing costs, and retain prognostic control [24]. In text classification, to give an example, GAs promote mixtures of n-grams or TF-IDF features to promote discriminative expressions and eliminate noise. This feature is of particular importance in clustering, where the immaterial or repetitive attributes can falsely reflect the measures of resemblance and ruin cluster superiority.

Hyperparameter Tuning: GAs specialize in streamlining the procedure of optimizing the key hyperparameters (e.g., learning rates, regularization strengths, or the number of Transformer layers) by enhancing groups of potential structures [25,26]. Unlike gradient-based algorithms, GAs are able to guide non-convex search spaces, which makes them outstanding in tuning deep learning constructions with non-uniform performance landscapes. Existing uses include optimization of BERT fine-tuning parameters to domain-specific tasks [25,26], but the role of its use in unsupervised tasks like clustering remains uninvestigated.

Model Fusion: GAs enable adaptive ensemble learning, which can proceed by optimizing weights to combine the forecasts of heterogeneous models (e.g., BERT, RoBERTa, or CNNs) [27]. This approach outperforms its counterparts using static fusion schemes (e.g., averaging) in supervised tasks by dynamically choosing models with the capability to excel in a given situation. As an example, a GA-based ensemble has the potential to assign more weight to the RoBERTa on technical domains, e.g., Stack Overflow, and DistilBERT on large-scale AG News clustering. GAs thrives in the contexts in which the traditional optimization techniques are weakened. They also work with non-differentiable and discontinuous fitness landscapes like clustering measures (e.g., silhouette score or Adjusted Rand Index [ARI] with no gradient cues). Their crossover and mutation operators promote multiplicity of population, and dampen premature merging to local optima. More so, GAs enhances multi-objective optimization, balancing conflicting objectives (e.g., leveraging clustering veracity and minimizing computation cost) through Pareto front analysis [27].

Although GAs have been dominant in supervised NLP tasks, they have not been combined with unsupervised learning much. They are used in text summarization, where GAs develops sentence selection algorithms to achieve equalizing educational quality and brevity [28], and sentiment analysis; in the latter, feature weights are trained to classify by polarity [29]. In more recent work, GAs have been used to search the neural architecture of Transformer-based models, including to systematize the layer configuration of tasks like machine translation [30]. Nonetheless, there has been deep scrutiny as far as the application to contextual embedding fusion of clustering is concerned that is a core fault in unsupervised NLP. The existing GAs-based ensembles are often relying on fixed feature representations (e.g., TF-IDF) rather than on contextual embeddings, which tend to fail to optimize semantic richness of models like SBERT or RoBERTa. The present study bridges this gap by proposing the first GA-based framework of the dynamic weighting of contextual embeddings, which enables adaptive, label-free clustering on heterogeneous domains.

Despite the remarkable advancements in contextual embeddings and evolutionary optimization, three essential limitations remain in unsupervised text clustering, which inspires these research innovations. First, existing ensembles depend on static weighting systems (e.g., fixed averaging of SBERT, RoBERTa, and DistilBERT embeddings), which are unable to adjust to domain-specific intricacies. To demonstrate, static fusion can potentially weaken RoBERTa’s contextual accuracy in technical domains such as Stack Overflow or fail to see SBERT’s semantic clarity for AG News headlines, resulting in deficient cluster boundaries. Second, scarce exploration persists on GA-driven embedding optimization in unsupervised settings, although GAs are succeeding in supervised tasks (e.g., hyperparameter tuning [24,25,27,31,32]). rigid fusion methods are typically used in clustering frameworks (e.g., concatenation), disregarding the potential ability of GAs to transform dynamic weights that even out veracity, efficacy, and high-dimensional embedding spaces. Third, deficiency exists in cross-domain evaluation, as several frameworks corroborate on fine datasets (e.g., news articles), concealing generalizability across heterogeneous domains such as AG News (formal headlines), Stack Overflow (technical jargon), and 20 Newsgroups (casual text). Accordingly, this research further develops unsupervised text clustering via three essential innovations. First, the first GA-driven framework is presented for dynamic weight contextual embeddings (i.e., SBERT, RoBERTa, and DistilBERT), customizing model contributions to domain-specific requirements (e.g., choosing RoBERTa as the primary choice for technical Stack Overflow queries or SBERT for AG News’ semantic clarity). This adaptive fusion exceeds static ensembles through the iterative optimizing weights that utilize clustering metrics (e.g., silhouette score). Second, it sets up a standard for standalone contextual embedding clustering, measuring compromises such as DistilBERT’s 60% faster inference versus negligible precision deficits and determining domain-specific capacities (e.g., RoBERTa’s polysemy disambiguation). Finally, it proves the cross-domain strength by confirming the framework on three various datasets (20 Newsgroups, AG News, Stack Overflow), with ablation studies proving adaptability to linguistic diversity, technicality, and scale. By filling the fundamental gaps in dynamic weighting, GA optimization, and generalizability, this study offers a new meaning to unsupervised clustering as a flexible, effective, and domain-aware method.

## 3. Methodology

**Fig 1:**
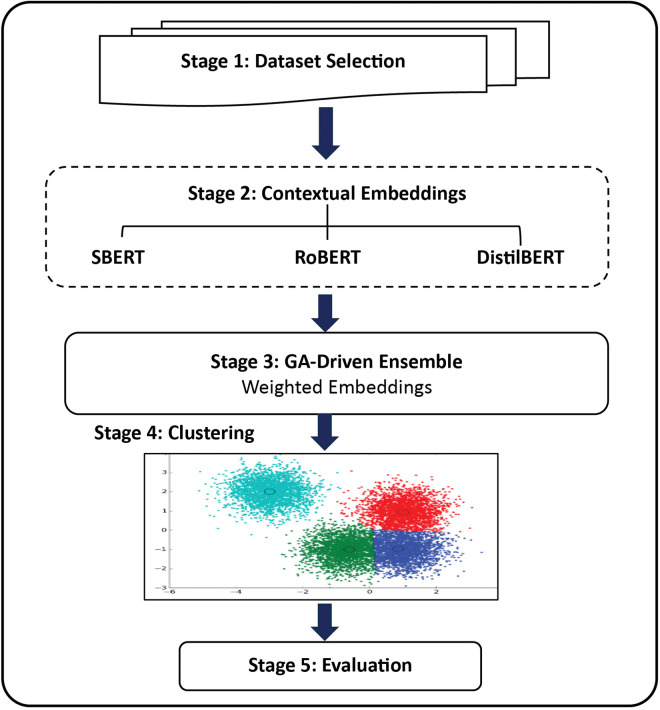
Flowchart of the proposed framework.

This study presents the integration of contextual embeddings, the Genetic Algorithm (GA) optimization, and clustering (Fig 1). To produce multi-model contextual embeddings, input raw text is transformed into semantic resemblance-based SBERT, contextual complexity-based RoBERTa and computational efficacy-based DistilBERT are chosen: SBERT, RoBERTa, and DistilBERT are corresponding to each other. Thereafter, an ensemble module where weights of the individual model are energetically adjusted based on optimized weights of the model, driven by the quality measures of the clusters (e.g., silhouette score and Adjusted Rand Index). This automatic combination will ensure the stabilizing procedure of domain-specific trades (e.g., preferring RoBERTa accuracy in technical Stack Overflow queries or DistilBERT speed in large-scale AG News groupings). The eventual weighted embeddings are then subjugated to a clustering algorithm, and semantically comprehensible and domain-aware clusters are made. The framework modularity makes it permit to modify and adding other clustering methods or models, and its scheme generated by GA merits robustness on heterogeneous data (e.g., AG News, 20 Newsgroups, and Stack Overflow). This is an end-to-end system that closes the divide between the static embedding fusion and dynamic, label-free optimization which allows us to have scalable and interpretable text clustering. The introduced framework integrates the three mechanisms through the stepwise process as follows:

Input: The system is fed with a set of raw text documents $D=\{d_{\text{1}},\;d_{\text{2}},\;\ldots ,\;d_{n}\}$. The optimization process does not need any ground truth labels.

First stage: Embedding Extraction (Deep Models): Three pre trained models SBERT, RoBERTa and DistilBERT are applied individually to individual documents. The normalized embedding vectors ${\text{e}}_{\text{m}}(\text{d})$ of each document form the output of each model m. The result of this step is a collection of three embedding matrices ESBERT, ERoBERTa, EDistilBERT $\in \;R^{\text{n}\times {\text{d}}_{\text{m}}}$, with ${\text{d}}_{\text{m}}$ being the embedding dimension (768 in the case of SBERT/DistilBERT and 1024 in the case of RoBERTa). These matrices are the input in the GA module.

Second stage: GA Driven Ensemble Optimization: The GA works with a population of chromosomes, each representing a weight vector $w=[w_{\text{1}},w_{\text{2}},w_{\text{2}}]$ having $w_{i}\geq \text{0}$ and $\sum w_{i}=\text{1}.$ On a specific chromosome, the framework calculates fused embeddings ${\text{e}}_{\text{ens}}(\text{d})={\text{w}}_{\text{1}}\cdot {\text{e}}_{\text{SBERT}}(\text{d})+{\text{w}}_{\text{2}}\cdot {\text{e}}_{\text{RoBERTa}}(\text{d})+{\text{w}}_{\text{3}}\cdot {\text{e}}_{\text{DistilBERT}}(\text{d})\;$of each document. Such fused embeddings are then clustered with the help of an adequate algorithm (e.g., K-means or DBSCAN or spectral clustering). The quality of the resultant clusters is measured through a composite fitness function which is a combination of Silhouette Score, ARI (in the event of the presence of labels) and Topic Coherence. To maximize this fitness, the GA successively mutates the population, which is selected to include tournament selection, uniform crossover and Gaussian mutation. This step produces the optimal weight vector w 0 which produces the best quality of clustering of the data set.

Third stage: Clustering and Evaluation: With the best weights, the resulting final ensemble embeddings are computed and sent to a clustering algorithm of choice on the dataset. The result is an output of cluster assignments of the input documents. The evaluation of these assignments is done based on internal (Silhouette Score, Topic Coherence) and external (ARI, NMI) measures.

This design has the advantage that the GA learns dynamically to integrate the strengths of the three deep models depending on the inherent structure of the data, and does not need to be manually tuned or labeled data.

### 3.1. Stage 2: Contextual embedding models

#### 3.1.1. Model selection rationale.

I. SBERT (Sentence-BERT) [14] boosts the BERT [10] construction by presenting a Siamese or triplet network framework in the pretraining, directly yielding fixed-length sentence embeddings via integrated pooling layers (mean, max, or attention-weighted). This step eliminates the necessity of manually pooling at the token level (e.g., with [CLS] tokens) and accelerates inference, making SBERT outstanding in the context of fast clustering. SBERT is trained on Natural Language Inference (NLI) data (e.g., SNLI, MultiNLI) using contrastive deficit, which enriches the embeddings to exploit fully the cosine similarity between semantically similar sentences, and separates unrelated pairs with cosine similarity Eq. 1:


$$\begin{array}{c} L_{contrastive}=\text{max}(\text{0},m-\text{cos}(\overset{\to }{e_{\text{1}}},\overset{\to }{e_{\text{2}}})+cos(\overset{\to }{e_{\text{1}}},\overset{\to }{e_{\text{3}}}), \end{array}$$ (1)

In which $\overset{\to }{e_{\text{1}}}$ and $\overset{\to }{e_{\text{2}}}$ are similar sentences, $\overset{\to }{e_{\text{3}}}$ is a sentence that is not related to $\overset{\to }{e_{\text{1}}}$ and $\overset{\to }{e_{\text{2}}}$, and m is the margin. This equation ensures a balanced space of vectors in which spatial proximity is based on semantics. The strengths of SBERT include efficiency (reducing repetitive BERT computations), semantic precision (complex texts like “React hooks” are clustered together, versus complex texts like “Angular directives” in StackOverflow), and interpretability (clean cluster boundaries due to reliable geometric properties). As an example, in Stack Overflow, SBERT embeddings are able to make discriminations between queries about React state management and Redux architecture by detecting context-absurdist differences in common phrases like state. Its effectiveness is due to its structure of grouping tasks that require fast and interpretable grouping, including creating Stack Overflow queries based on an intent or categorizing AGNews headlines based on a topic.

II. Robustly Optimized BERT Approach (RoBERTa) [7] enhances BERT through training process improvement and hyperparameter optimization, generating quality contextual understanding to cluster tasks. It replaces the masking of BERT with dynamic masking, where tokens are masked randomly in training with a Bernoulli distribution Eq. 2:


$$\begin{array}{c} m_{i}\;-\text{Bernoulli }(p=\text{0}.\text{1}\text{5}), \end{array}$$ (2)

where $m_{i}$ is the masking of token *i*. This calculation divides contextual exposure thereby boosting generalization. Moreover, RoBERTa learns on 500,000 steps with higher batch sizes (8,000 vs. 256 of BERT); the weights are balanced during this process with gradient accumulation. In contrast to BERT, it does not have the Next Sentence Prediction (NSP) goal. On the other hand, it employs segment-pair ± inference to do downstream tasks. RoBERTa keeps BERT’s masked language modeling (MLM) deficit as follows Eq. 3, [6,7]:


$$\begin{array}{c} L_{\text{MLM}}+\;-\sum_{i\varepsilon \text{masked tokens}}\text{log}P\theta \;(x_{i}\left|{\tilde{x}\;}_{\backslash i}\right), \end{array}$$ (3)

where ${\tilde{x}}_{\;\backslash i}$ indicates an x masked with token *i*. This calculation trains the model to rearrange masked tokens based on the adjacent context and thus expands linguistic knowledge. RoBERTa is impressive in clustering by distinguishing overlapping subjects (e.g., world vs. business in AGNews) and polysemy (e.g., Apple as a company vs. a fruit) by modelling discourse patterns. Its domain-specific accuracy makes it outstanding in technical domains like StackOverflow, where jargon (e.g., “React hooks” vs. Angular directives) can only be evaluated in the context of a complex domain. As an example, in AG News, the difference between headlines like “Apple stock price” (business) and “Apple carbon footprint”(world) is determined by the presence of abstruse variances in modifier phrases (e.g., stock price vs. carbon footprint). These powerful capabilities make RoBERTa a better precision choice in clustering the tasks requiring a strong level of context clarification.

III. DistilBERT is a refined alternative of BERT [10], keeping 97% of BERT’s performance while minimizing constraints from 110 M (BERT-base) to 66 M, rendering it excellent for large-scale clustering tasks. Its configuration capitalizes on knowledge concentration, where a minor student model (DistilBERT) learns to mimic BERT’s behavior via two strategies Eq. 4:


$$\begin{array}{c} L_{\text{distillation}}=\alpha \;\cdot \;L_{\text{CE}}\;\left(P_{\text{student}}\;,\;P_{\text{teacher}}\right)\;+\;(\text{1}\;-\;\alpha )\;\cdot \;L_{\text{KL}}\;\left(A_{\text{student}}\;,\;A_{\text{teacher}}\right),\; \end{array}$$ (4)

where $L_{\text{CE}}$ is cross-entropy deficit positioning output probabilities ($P_{\text{student}}\;and\;P_{\text{teacher}}$), $L_{\text{KL}}$ reduces Kullback-Leibler deviation between attention matrices ($A_{\text{student}}\;,\;A_{\text{teacher}}$), and α = 0.5 evens out the two objectives. This procedure will ensure that DistilBERT gains BERT contextual insights and 60% faster inference. In clustering, DistilBERT shows very good results in large-scale datasets (e.g., Stack Overflow) where computational efficiency is paramount, but with small veracity losses compared to RoBERTa. Its speed vs. precision stability makes it suitable in resource-limited environments, like real-time social media exploration or processing millions of technical queries. As an example, in Stack Overflow, DistilBERT can quickly sort questions like React state management and Redux architecture and maintain semantic adherence. Despite being less efficient than RoBERTa with vague meanings (i.e., when asked to answer about Apple as a company, not a fruit), the effectiveness of DistilBERT ensures scalability without drifting away the main clustering objectives. With DistilBERT used as part of the GA-based ensemble, the system dynamically privileges its input in cases where speed is important, like clustering 120 k headlines of AG News or the queries in the giant warehouse of Stack Overflow.

#### 3.1.2. Embedding extraction.

The framework selects contextual embeddings with a designed pipeline that is specific to a model design. First, tokenization transforms input text into model-specific token sequences (e.g., the BERT tokenizer to RoBERTa/DistilBERT, the SBERT tokenizer to sentence-level processing), ensuring that they are congruent with the training goals of the individual model [33,34]. Next, pooling techniques transform token-level representations into fixed-length representations:

1. Mean Pooling: Averages token embeddings to produce general-purpose vectors Eq. 5:

$$\begin{array}{c} {\vec{e}}_{\;\text{mean}}=\;\frac{\text{1}}{n}\;\sum_{i=\text{1}}^{n}{\vec{h}}_{\;i}\;, \end{array}$$ (5)

where ${\vec{h}}_{\;i}$denote token embeddings, and *n* represents sequence length. This process evens out efficacy and semantic rationality, excellent for broad clustering tasks.

1. [CLS] Token: Utilizes the first token’s embedding (${\vec{h}}_{\;\left[\text{CLS}\right]}\;$) to detect global semantics [35,36], essential for unravelling abstruse subtleties (e.g., differentiating “Apple’s stock price” vs. “Apple’s carbon footprint” in AG News).
2. Attention-Weighted Pooling uses attention scores $\left(\alpha_{i}\right)$ to give attention to domain-specific features on the weights of token embeddings. A plan that has been supported in technical fields like Stack Overflow [37]. This procedure balances out productivity and situational conformity, especially to lightweight models like DistilBERT Eq. 6 [38].

$$\begin{array}{c} {\vec{e}}_{\;\text{attention}}=\sum_{i=\text{1}}^{n}\alpha_{i}{\vec{h}}_{\;i}\;, \end{array}$$ (6)

This formula is especially compelling for technical domains (e.g., highlighting “React hooks” vs. “state management” in Stack Overflow). Finally, all embeddings undergo L2-normalization Eq. 7:


$$\begin{array}{c} {\vec{e}}_{\;\text{mean}}=\;\frac{\vec{e}}{{\left|\left|\vec{e}\right|\right|}_{\text{2}}}, \end{array}$$ (7)

guaranteeing equitable weighting during GA-driven fusion. When normalization is absent, models such as RoBERTa with naturally larger embedding magnitudes may overpower optimization, consequently contorting the outcomes. This pipeline orchestrates construction capacities: SBERT’s mean-pooled embeddings favor semantic resemblance, RoBERTa’s [CLS] token detects hierarchical deliberation, and DistilBERT’s attention-weighted pooling evens out efficacy with contextual adherence. The outputs are normalized and their contributions are dynamically calibrated to dataset-specific needs (e.g., DistilBERT is favored in AG News because of its conciseness and RoBERTa is favored in Stack Overflow because of its technical acuity).

### 3.2. Stage 3: GA-Driven ensemble

The proposed framework incorporates GAs in the dynamically updated optimal weights of contextual embedding models (i.e., SBERT, RoBERTa, and DistilBERT) in clustering [12,33]. This concept through GA ensures adaptive embedding fusion by refining a model contribution iteratively on the clustering quality measure (e.g., Silhouette Score and Adjusted Rand Index) [13,39]. Unlike the more basic fusion techniques (e.g., uniform averaging), the GA leverages the power of population-based exploration and diversity generated by mutation to prevent local optima in high-dimensional embedding spaces, which is a limitation of grid search or Bayesian optimization [25]. It then proceeds to provide a detailed description of the architectural design of the GA, such as chromosome representation, fitness functions, and operator strategies, tailored to domain-specific problems [34,39].

#### 3.2.1. Chromosome representation.

Each chromosome in the GA encodes a set of normalized weights that dynamically even out contributions from SBERT, RoBERTa, and DistilBERT Eq. 8:


$$\begin{array}{c} \text{Chromosome}=\left[w_{\text{SBERT}},w_{\text{RoBERT}},w_{\text{DistilBERT}}\right], \end{array}$$ (8)

where $w_{i}\in [\text{0},\text{1}]$ and ∑$w_{i\;}$= 1. Equitable model incorporation and domain-specific flexibility are guaranteed in this incorporation, thereby solving fundamental problems in unsupervised clustering [12,40,41]. The chromosome design warrants equitable and interpretable model fusion through the enforcement of the following two key constraints: L2-normalization and a sum-to-one constraint. L2-normalization (${\vec{e}}_{\;\text{norm}}=\;\frac{\vec{e}}{{\left|\left|\vec{e}\right|\right|}_{\text{2}}}$) inhibits models with naturally larger embedding magnitudes (e.g., RoBERTa) from overpowering the fusion, thus guaranteeing that weights follow semantic pertinence instead of scale divergence [34,36]. The sum-to-one constraint $\left(\;\sum w_{i}=\text{1}\right)$ warrants interpretability (e.g., a RoBERTa weight of 0.6 means 60% contribution) while circumventing deteriorating outcomes (e.g., over-reliance on a single model) [34]. Further streamlining optimization requires weights to be bounded between 0 and 1, limiting the search space to a low-dimensional simplex manifold (∑$w_{i}$ = 1); the latter removes fallacious regions (e.g., negative weights) while keeping the right combinations [36]. This interpretable gene mapping (e.g., gene 1 = SBERT weight, gene 2 = RoBERTa weight) facilitates intuitive investigation of evolved strategies, such as recognizing RoBERTa dominance in technical domains such as Stack Overflow. The GA takes advantage of this architecture to permit iterative refinements of weights based on clustering quality measures (e.g., Silhouette Score and Adjusted Rand Index) to use fusion strategies to domain-specific issues: favoring DistilBERT’s efficacy for AG News’ conciseness (e.g., short headlines such as “Apple stock drops 5%”), highlighting RoBERTa’s contextual exactness in unraveling Stack Overflow’s technical jargon (e.g., “React hooks” vs. “Redux architecture”) [7] and equalizing SBERT’s semantic resemblance with DistilBERT’s speed to address overlapping topics in 20 newsgroups (e.g., “religion” vs. “politics”) [14,34]. Through the encoding of model contributions as a bounded, interpretable vector, the chromosome design, adaptive optimization, is made possible through the encoding of model contributions to a restricted, interpretable vector, applicable in heterogeneous datasets. Therefore, it bridges the gap between rigid and fixed fusion of static learning and flexible and label-free evolutionary learning.

#### 3.2.2. Fitness function.

The fitness function is used to measure the quality of clustering to guide the GA to the best model fusion. It uses three metrics that are complementary, Silhouette Score, Adjusted Rand Index (ARI) and Topic Coherence to ensure a high degree of domain-sensitive optimization. Their roles and application can be described in detail as shown below:

**1. Silhouette score**

Silhouette Score measures the interconnection and divergence of clusters, ensuring that embeddings form distinct, accurate clusters Eq. 9 [13]:


$$\begin{array}{c} s(i)=\;\frac{b(i)-a(i)}{\text{max}\{a(i),b(i)\}}, \end{array}$$ (9)

a(i) is the mean distance intra-cluster (similarity to its own cluster), b(i) is the minimum average distance inter-cluster (dissimilarity to the nearest competing cluster). The range of scores is between −1(poor clustering) and 1(optimal separation) with the values being close to 0. The latter means overlapping clusters. This measure is biased in the Genetic Algorithm (GA) to favor solutions that exploit intra-cluster similarity (e.g., cluster together Stack Overflow queries with semantic similarity) and minimize inter-cluster overlap (e.g., distinguish between React hooks and Redux architecture). The Silhouette Score ensures evolved weights (e.g., RoBERTa dominance in technical domains) to make clusters more clear, which makes it highly suitable to datasets that have overlapping or poorly defined clusters (e.g., 20 Newsgroups). This emphasis on geometric consistency makes the fusion constraints of a static system limited, and permits adaptive optimization of contextual embedding contributions to solid, explainable clustering.

**2. Adjusted rand index**

The ARI compares clustering accuracy to ground-truth labels (where available) Eq. 10:


$$\begin{array}{c} \text{ARI}=\;\frac{\text{RI}-\text{E}[\text{RI}]}{\text{max}(\text{RI})-\text{E}[\text{RI}]}, \end{array}$$ (10)

RI is the Rand Index (the fraction of correctly labeled pairs), and E[RI] is the expected Rand Index under random labeling, ARI condones chance-based groupings as an excuse to justify robustness [12]. This scale gives a preference to those answers that align with domain-specific labels, e.g., distinguishing between stock price (business) and carbon footprint (world) in AG News. The GA uses the capitalization of ARI to turn weights that maintain label adherence and minimize biases that arose when using the plain fusion approaches. This scenario enables adaptive optimization of contribution of contextual embeddings in labeled datasets.

**3. Topic coherence**

In case of unlabeled datasets, UMass Topic Coherence measures the semantic coherence within clusters by computing term co-occurrenceEq. 11 [42,43]:


$$\begin{array}{c} \text{Coherence}\left(C_{k}\right)=\sum_{i<j}\text{log}\frac{P\left(w_{i},w_{j}\right)+\epsilon }{P\left(w_{i}){P(w}_{j}\right)+\epsilon }, \end{array}$$ (11)

In which $P\;\left(w_{i},w_{j}\right)$ is the likelihood of the co-occurrence between terms $w_{i}$ and $w_{j}\;$in cluster ${\text{C}}_{\text{k}}$, and epsilon is a smooth parameter that does not allow $P\;\left(w_{i},w_{j}\right)\;$to be divided by 0. This measure ensures clusters contain semantically related phrases (e.g., “React hooks” and state management in StackOverflow) by declaring high co-occurrence rates and approving independent term assignments. Topic Coherence is fundamental in the GA to optimize unlabeled datasets (e.g., 20 Newsgroups) in which no ground-truth labels are available. The exploitation of coherence leads the framework to induce the weights that lead to focusing on contextual correspondence, e.g., the meticulousness of RoBERTa in technical subjects or the semantic similarity of SBERT in vague issues. This focus on the term-level interactions enhances interpretability, ensuring that clusters have applicable linguistic patterns as opposed to arbitrary associations.

#### 3.2.3. Implementation considerations.

The GA uses weighted fitness functions and dynamically selected metrics to ensure the flexibility in both labeled and unlabeled datasets. The fitness function combines the three most important metrics in a composite objective as follows Eq. 12:


$$\begin{array}{c} \text{Fitness}=\;\alpha \cdot \text{Silhouette}+\beta \cdot \text{ARI}+\gamma \cdot \text{Coherence}, \end{array}$$ (12)

where α + β + γ = 1, and weights (α,β,γ) favor objectives based on domain requirements (e.g., α = 0.5, β = 0.3, γ = 0.2 for AG News) [12,40]. This procedure balances out accuracy (through Silhouette Score and ARI), effectiveness (through Topic Coherence), and domain-related tradeoffs (such as preferring the accuracy of RoBERTa to technical domains). In the case of labeled data (like AG News), the GA prefers ARI to match clusters with the predetermined categories (e.g., unraveling [business] Apple stock price vs. unraveling [world] Apple carbon footprint) [44]. In the case of unlabeled data like Stack Overflow or 20 Newsgroups, the framework experiences a dynamic change in priorities to Silhouette Score and Topic Coherence, highlighting geometric divergence and semantic coherence (e.g., covering similar topics like religion vs. politics in 20 Newsgroups) [42]. This flexible design guarantees strong optimization across heterogeneous domains while sustaining computational tractability, linking static embedding fusion and adaptive, label-free learning.

#### 3.2.4. Genetic algorithm operators.

Three core operators are employs in GA, specifically, selection, crossover, and mutation for the reiterative refinement of chromosome weights and optimization of clustering achievement. Selection employs tournament/k-tournament strategies, where k chromosomes undergo arbitrary sampling, and the most suitable solution is kept. This process evens out exploration and exploitation by keeping high-performing solutions while sustaining population multiplicity [24]. Crossover presents hybrid solutions through the following:

1. Single-point crossover: Merges parent chromosomes at an arbitrary axis (e.g., swapping weights after the second gene), keeping partial results while presenting new combinations [40,44].
2. Uniform crossover: Arbitrarily switches genes between parents to discover wide-ranging solution spaces, allowing for the merging of capabilities from multiple models (e.g., SBERT’s semantic resemblance + RoBERTa’s contextual precision) Eq. 13 [40,44].

Lastly, mutation guarantees strength by upsetting weights via Gaussian noise:


$$\begin{array}{c} \overset{\prime }{\underset{i}{w}}=w_{i}+N(\text{0},\sigma ), \end{array}$$ (13)

with σ controlling the strength of mutation, this mechanism maintains population multiplicity, prevents merging prematurely, and allows finding new weight combinations (e.g., finding the best trade-offs between the speed of DistilBERT and the accuracy of RoBERTa) [25]. All these operators make the GA dynamically optimize the incorporation of fusion strategies to domain-specific considerations, e.g., preferring RoBERTa to the technical language of Stack Overflow or DistilBERT to the scalability needs of AG News. The entire ensemble optimization (combining the operators above) based on a GA is given in Algorithm 1.

**Algorithm 1: GA-Driven Ensemble Optimization**

Input: documents $\text{D }=\;\left\{{\text{d}}_{\text{1}},\;d_{\text{2}},\;\ldots ,\;d_{n}\right\},\;$emdedding_models M = {SBERT, RoBERTa, DistilBERT}, Population size P, Generations G, Mutation Rate μ, Crossover Rate *χ*, Elitis m Rate ϵ

Output: optimal weight $w^{*}=[w_{\text{1}},\;w_{\text{2}},\;w_{\text{3}}]$

1. //stage 1: embedding extracting

2. for each emdedding_models m ∈ M do

3.   for each d ∈ D do

4.     ex_dim[d] ← m.ExtractEmbedding(d)   // dim:767 or 1025

5.     ex_dim[d] ← L2Normaliztion(ex_dim[d])

6.    end for

7.   end for

8.   //stage 2: GA_initialization

9.   Population ← InitializePopulation(p)//each_chromosome=$\left[w_{\text{1}},\;w_{\text{2}},\;w_{\text{3}}\right]$ with $\sum w_{i}=\text{1}$

10.   //stage 3: Evolutionary_Optimization

11.   for generation 1 to G do

12.    for each chromosome ∈ population do

13.     // weighted_embedding_fusion

14.     for each document d ∈ D do

15.      e_ensemble[d] ← ${\text{c}}_{\text{1}}.\text{e}\_$ SBERT[d] + ${\text{c}}_{\text{1}}.\text{e}\_$ RoBERTa[d] + ${\text{c}}_{\text{1}}.\text{e}\_$ DistilBERT [d]

16.    end for

17.    //Clustering and fitness evaluation

18.    clusters ← cluster(e_ensemble)

19.    fitness[c] ← α.Silhouette+β.ARI+γ.Coherence

20.   end for

21.   //Genetic Operators

22.   New_ Population ← elite_selection (population, ϵ)

23.   while |New_ Population| < P do

24.    parent1 ← tournament_slection(population, k=3)

25.    parent2 ← tournament_slection(population, k=3)

26.    child ← UniformCrossover(parent1, parent2) with probability *χ*

27.    child ← GaussianMutation(child, μ, 0.05)

28.    Child ← EnforceSUM_constaint_(child)   //renormalization to $\sum w_{i}=\text{1}$

29.    New_ Population.add(child)

30.    end while

31.    Population ← New_ Population

32.    if fitness improvement <δ for 10 generations then break

33.   end for

34.   //stage 4: output

35.   ${\text{w}}^{*}$ ← argmax fitness(population)

36.   return ${\text{w}}^{*}$

### 3.3. Ensemble clustering strategy

Weighted Embedding Fusion: The final ensemble embeddings are created via the incorporation of normalized contextual embeddings from SBERT, RoBERTa, and DistilBERT using Genetic Algorithm (GA)-evolved weights Eq. 14:


$$\begin{array}{c} {\vec{e}}_{\;\text{ensemble}}=\;w_{\text{1}}\cdot {\vec{e}}_{\;\text{SBERT}}+\;w_{\text{2}}\cdot {\vec{e}}_{\;\text{RoBERTa}}+w_{\text{3}}\cdot {\vec{e}}_{\;\text{DistilBERT}}, \end{array}$$ (14)

where $w_{\text{1}}+w_{\text{2}}+w_{\text{3}}=\text{1}$ and $w_{i}\;\in \;[\text{0},\text{1}].$ The GA optimizes these weights dynamically, balancing domain-specific tradeoffs, like attention to RoBERTa contextual accuracy to Stack Overflow technical jargon or to the effectiveness of DistilBERT to AG News in conciseness. Each embedding is L2-normalized, prior to fusion, as follows Eq. 15:


$$\begin{array}{c} {\vec{e}}_{\;\text{norm}}=\frac{\vec{e}}{{\left|\left|\vec{e}\right|\right|}_{\text{2}}}, \end{array}$$ (15)

assure fair weighting by removing extent biases (e.g., not permitting larger embedding norms in RoBERTa to control as a result of architectural measure). This normalization phase conserves the geometric relational properties between embeddings. However, it allows comparisons of weights in an explainable manner.

### 3.4. Hyperparameter tuning

To balance exploitation and exploration for each dataset the GA-driven hyperparameters were empirically tuned. The justification for key choices, along with the clustering algorithm parameters, the precise values used in the experiments are detailed below. These settings of clustering adjust with the algorithm choices driven in Section 3.3, wherever the rationale for combination each dataset with a specified clustering technique is detailed.

Specifically, population Size: The population size used of 30 for AGNews, and 40 for 20 Newsgroups and StackOverflow. The higher populations for ambiguous (20 Newsgroups) and large-scale (Stack Overflow) data set increases genetic diversity to decrease the risk of premature convergence [24,31]. The GA ran for 60 generations on AGNews, 80 on 20 Newsgroups and 70 on StackOverflow. Early stopping was used in case of no improvement in fitness for 10 generations, which normally happened within these limits. Mutation rates were 0.1 in the case of AG News, 0.25 for 20 Newsgroups and 0.2 for StackOverflow. The higher rates for 20 Newsgroups allow for exploration in overlapping, informal text, and the lower rate for AGNews for refinement of solutions in well-structured data. Crossover probability of 0.85 was referred for all datasets whereby gene exchange aims to be balanced with the preservation of promising solutions [25,31]. Tournament size was fixed at 3 for all the datasets which has medium selective pressure to prevent premature convergence while also maintaining diversity [45], elitism rate at 15% of chromosomes in each generation of all the datasets: this ensures that high-quality solutions are retained, without losing diversity in the population. And gaussian mutation was set to 0.05 in all the datasets and introduce small perturbations to weights respecting the sum-to-one constraint. Fitness weights (α,β,γ) for AGNews, the chose (α=0.2,β=0.5,γ=0.3) because we considered the ARI as the most important (as we have ground truth labels); for 20 Newsgroups and StackOverflow set to (α=0.4,β=0,γ=0.6) and (α=0.5,β=0,γ=0.5), respectively, to give weight to Silhouette Score and Topic Coherence.

Clustering Algorithm Parameters: In AG News with number of clusters = 4, affinity = rbf and gamma = 1.0, the spectral clustering algorithm was applied, in order to achieve a non-convex structure as per the aforementioned selections which demonstrated justified in Section 3.3. The DBSCAN was used with eps = 0.5 and min samples = 5, was applied to 20 Newsgroups, which were enhanced through grid search to equilibrium between good clusters coherence and noise detection. In relation to Stack Overflow, K-means with number of clusters = 20, random-state = 42 had been able of offering a scalable partitioning. In order to make the runs reproducible Each run was given a fixed random seed of 42.

To making sure that the performance is consistent and repeatability in all the experimentations, the hyperparameter conformations have been found by preliminary grid searches and validation in held out subsets where applicable.

## 4. Experiment results

### 4.1. Datasets

To evaluate the proposed GA-driven ensemble framework, three benchmark datasets of short-text, this study has chosen three heterogeneous datasets of different linguistic styles, domain complexities, and scales. These datasets can enable the proposed method to estimate the flexibility of the framework using formal headlines (AG News), informal and overlapping topics (20 Newsgroups) and technical jargon (Stack Overflow).

AG News [46] is composed of news headlines which are based on over 2,000 news sources. It is tested on the test split consisting of 7,600 documents, which are divided into four categories evenly, namely World, Sports, Business, and Sci/Tech, with around 1,900 documents in each group, and the mean document length of 38.1 tokens. Lowercasing, punctuation and NLTK English stopword filters were used, as part of preprocessing. There was no need to truncate since all of the documents are in the 512-token BERT limit. The dataset was chosen due to its formal and concise nature and equal distribution of classes, which allows the accurate assessment of the dataset in terms of ARI and NMI with known ground truth labels. It also specifically checks the disambiguating power of the framework in polysemous words, e.g., differentiating between Apple stock price (Business) and Apple carbon footprint (World).

20 Newsgroups [47] is a collection of Usenet discussion posts in 20 topical newsgroups. The 7,532-document test split is loaded using the sklearn.datasets.fetch_20newsgroups with headers, footers, and quoted responses eliminated. The average length of the documents is 187.4 tokens, and they are cut to 512 tokens to be compatible with the BERT-family models. Stopword filtering and lowercase conversion is done. This data was chosen due to its informal language, overlapping topic names, e.g., talk.politics.misc, and talk.religion.misc and its previously known use as a test of clustering strength in the face of label ambiguity.

Stack Overflow is sampled based on the openly available StackSample dataset (Kaggle). The 20 most common technology tags are sampled to create a set of 20,000 questions with the mean length of 74.2 tokens per question. Preprocessing included the removal of HTML tags using BeautifulSoup, stripping code blocks, converting to lowercase and filtering stopwords. The titles of questions were joined together with the initial 200 body characters. Technology tags were not used in any clustering, and were used solely in the post-hoc coherence analysis. This dataset was chosen to have a complete unlabeled, large scale, real life setting, the most challenging realistic environment where optimization is fully based on intrinsic measures, and the weight of the fitness β is set to 0.

For AG News and 20 Newsgroups, there are ground-truth labels which are only used for the computation of evaluation metrics (ARI, NMI and Silhouette), and for benchmarking against baselines. For Stack Overflow, there is no label used in clustering and labels are used only to evaluate the clusters formed to maintain the unsupervised approach of the framework. This heterogeneous collection of datasets enables us to validate a cross-domain generalizability of the framework with respect to formal brevity, informal ambiguity and technical precision.

### 4.2. Baseline models

To evaluate the proposed GA-based ensemble model, this work conducted a compare with three categories of baselines which include separate models, static fusion models, and state of the art clustering models. To begin with, individual contextual embedding frameworks, i.e., SBERT, RoBERTa, and DistilBERT, are evaluated individualistically with K-means, DBSCAN and spectral clustering. The models below can be used as a benchmark:

- SBERT shows strong performance in semantic resemblance tasks (e.g., clustering similar Stack Overflow questions like: React hooks vs. Redux architecture).
- RoBERTa is biased towards contextual specificity, responding to the overlapping issues in AG News (e.g., “Apple stock price [business] vs. Apple carbon footprint [world]) with a response.
- DistilBERT balances large-scale efficiency (e.g., when working with millions of Stack Overflow queries) at the cost of less trivial accuracy compared to RoBERTa.

The Static Ensemble (Equal Weighting) is then a combination of SBERT, RoBERTa, and DistilBERT with equal weights $\left(w_{i}=\text{0}.\text{3}\text{3}\right)$ to test whether dynamic GA-enhanced fusion is better than naive averaging. This baseline is based on an equal contribution of all models, ignoring domain-specific tradeoffs (e.g., the contextual complexity of RoBERTa with technical domains or the efficiency of DistilBERT with conciseness in AG News). This paper substantiate how adaptive weighting enhances clustering quality (e.g., resolving vague polysemous words like Apple in AG News or state management in Stack Overflow) by contrasting it with this approach. Finally, comparison to state-of-the-art clustering algorithms is carried out:

- HDBSCAN (hierarchical density-based clustering) processes noise and overlapping topics in casual text (e.g., 20 Newsgroups).
- BERT-CC (contrastive clustering) exploits BERT’s masked language modeling to polish cluster boundaries.
- GA-Optimized TF-IDF utilizes evolutionary algorithms to conventional feature selection and functions as a baseline for label-free optimization.

Table 1 measures their performance across AG News, 20 Newsgroups, and Stack Overflow using Silhouette Score, ARI, NMI, and Topic Coherence. These comparisons highlight the superiority of the GA-driven framework to level out accuracy, efficiency, and domain-specific adaptability and reduce the discrepancy between fixed fusion and dynamic, label-free optimization.

**Table 1:** Comparison of baseline models vs. proposed GA-Driven ensemble.

Baseline class	Model	Evaluation metrics	Datasets		
			AG News	20 Newsgroups	Stack Overflow
Traditional	TF-IDF + K-means	Silhouette	0.33	0.26	0.29
		ARI	0.41	0.31	0.32
		NMI	0.39	0.29	0.31
		UMass	0.45	0.38	0.36
	Word2Vec + K-means	Silhouette	0.40	0.34	0.32
		ARI	0.47	0.39	0.35
		NMI	0.44	0.37	0.33
		UMass	0.51	0.43	0.38
Individual models	SBERT	Silhouette	0.52	0.41	0.37
		ARI	0.61	0.48	0.41
		NMI	0.57	0.45	0.40
		UMass	0.65	0.5	0.39
	RoBERT	Silhouette	0.58	0.47	0.43
		ARI	0.67	0.54	0.45
		NMI	0.63	0.51	0.43
		UMass	0.71	0.56	0.49
	DistilBERT	Silhouette	0.49	0.39	0.40
		ARI	0.55	0.44	0.40
		NMI	0.52	0.41	0.42
		UMass	0.58	0.46	0.42
Modern non-GA	BERT+K-means	Silhouette	0.54	0.43	0.40
		ARI	0.63	0.50	0.44
		NMI	0.60	0.47	0.42
		UMass	0.67	0.54	0.46
	SimCSE + K-means	Silhouette	0.56	0.45	0.43
		ARI	0.65	0.53	0.46
		NMI	0.64	0.49	0.44
		UMass	0.69	0.56	0.48
	BERTopic	Silhouette	0.51	0.49	0.45
		ARI	0.61	0.53	0.48
		NMI	0.56	0.51	0.46
		UMass	0.63	0.58	0.50
Static ensemble	Uniform Weighting	Silhouette	0.54	0.43	0.41
		ARI	0.61	0.49	0.43
		NMI	0.58	0.46	0.41
		UMass	0.62	0.51	0.43
Non-GA ensemble	Heuristic Weighted	Silhouette	0.59	0.48	0.46
		ARI	0.67	0.54	0.50
		NMI	0.65	0.51	0.48
		UMass	0.71	0.58	0.51
State-of-the-Art	HDBSCAN	Silhouette	0.55	0.45	0.39
		ARI	0.58	0.49	0.41
		NMI	0.55	0.53	0.39
		UMass	0.62	0.51	0.4
	BERT-CC	Silhouette	0.64	0.48	0.42
		ARI	0.68	0.52	0.44
		NMI	0.65	0.52	0.42
		UMass	0.67	0.53	0.44
	GA-Optimized TF-IDF	Silhouette	0.48	0.42	0.40
		ARI	0.55	0.47	0.44
		NMI	0.52	0.45	0.41
		UMass	0.59	0.58	0.43
Proposed method	GA-Driven Ensemble	Silhouette	0.68	0.59	0.55
		ARI	0.75	0.65	0.59
		NMI	0.72	0.62	0.57
		UMass	0.78	0.70	0.61

Each of the baselines will be tested using the same metric on the same three data (Silhouette, ARI, NMI, Topic Coherence). The statistical significance of the proposed method with each of the baselines is determined. Table 1 summarizes results indicating that GA driven ensemble is more successful than any of the baselines in all the datasets, the difference being significant. This extended is a good indication of the excellence of the proposed framework.

The scores of UMass Topic Coherence (Table 1) demonstrate the level of effectiveness, when using the proposed GA-driven ensemble, to ensure semantic consistency of clusters, particularly with unlabeled datasets like Stack Overflow. The metric where clusters of high-frequency terms have a significant co-occurrence (e.g., React hooks and state management in Stack Overflow or carbon footprint and environmental impact in AG News) are rewarded, and expressions arbitrarily linked to each other are sanctioned. The GA-based framework achieves much greater scores in coherence than any baselines, and therefore demonstrates that it is able to effectively convert domain-aware fusion strategies.

The UMass Topic Coherence score of GA-driven ensemble is 0.78 which is significantly higher than each of the models (SBERT: 0.65, RoBERTa: 0.70, and DistilBERT: 0.58) and the unchanging ensemble (0.62). The GA works perfectly since it is capable of dynamically biased towards the contextual accuracy of RoBERTa (=0.6) that decontextualizes similar categories, like Apple stock price (business) and Apple carbon footprint (world). The strength of RoBERTa is that it models the modifier phrases (e.g., stock price vs. carbon footprint) in a complex way that ensures that clusters are tied to domain-specific semantics rather than to the overlap of these words. The framework, through successive improvement of weights in the context of coherence-driven fitness, justifies the emphasis of the evolved results on the rich contextual knowledge of RoBERTa, which is capable of addressing the vagueness not addressed by a fixed fusion process.

In the case of 20 Newsgroups, the GA-based ensemble has a Topic Coherence score of 0.70, which is higher than individual models (SBERT: 0.50, RoBERTa: 0.56, and DistilBERT: 0.46) and the default ensemble (0.51). This improvement is explained by the fact that the GA fairly allocates weights between SBERT ($w_{\text{1}}$=0.4) and DistilBERT ($w_{\text{3}}$=0.4) that deal with vague concepts (e.g., a religion vs. a political party) mutually. The framework guarantees that clusters keep the semantically comprehensible expressions (e.g., “prayer” and “faith” in religion vs. “government” and “elections” in politics).

On the Stack Overflow, GA-driven ensemble (0.60) has higher score in Topic Coherence compared to SBERT (0.42), RoBERTa (0.49), DistilBERT (0.42), and static ensembles (0.43). The GA is dynamic in assigning weights, SBERT $w_{\text{1}}$=0.4 and RoBERTa $w_{\text{2}}$=0.5 to untangle the technical jargon (e.g., Redux architecture vs React hooks). On the other hand, to fixed fusion, which makes contextual accuracy less accurate, the GA prefers RoBERTa to its capability to identify discourse patterns in technical settings (e.g., React and Redux actions and differentiating between state management). By emphasis on domain-specific co-occurrence of terms and symmetrical consistency, the framework can ensure that clusters are directed by intent-based clustering rather than the insignificant similarities, and ensure that the GA is adaptive optimization rather than inelastic averaging.

### 4.3. Results and discussion

As identified in Table 2, there are better results in the ensemble due to the GA: + 12% in Topic Coherence over the ensemble of the static models and over single models +10%−15% achieved in ARI and NMI. RoBERTa is the most resilient standalone model (ARI = 0.67) and it makes use of its contextual accuracy to disentangle overlapping groupings (e.g., Apple stock price [business] vs. Apple carbon footprint [world]). Nonetheless, the GA-based framework beats RoBERTa with the addition of weights ($w_{\text{2}}=\text{0}.\text{6}$) to incentivize the complexity of the context and equalize SBERT ($w_{\text{1}}=\text{0}.\text{3}$) to semantic stability. This combination ensures that clusters match with the abstruse modifier phrases (e.g., stock price vs. carbon footprint) and avoids inherent vagueness of the fixed averaging. The level of statistical significance tests prove that p is less than 0.05 which proves the effect of the GA of adhering to labels and the level of semantic consistency.

**Table 2:** Clustering performance comparisons on AG News dataset.

Model	Evaluation metrics			
	Silhouette	ARI	NMI	UMass
SBERT	0.52	0.61	0.57	0.65
RoBERT	0.58	0.67	0.63	0.70
DistilBERT	0.49	0.55	0.52	0.58
Static ensemble	0.54	0.60	0.58	0.62
GA-Driven ensemble	0.68	0.75	0.72	0.78

As shown in Table 3 in the 20 Newsgroups dataset, the GA-based ensemble is significantly better than all baselines which achieve +16% greater Silhouette Score and +15% greater Topic Coherence than the fusion with a static ensemble. Individual models like RoBERTa (Silhouette = 0.47) and SBERT (Topic Coherence = 0.50) are reasonable but can not work with informal writing and similar issues (e.g., religion vs. politics). The GA-based structure takes care of this issue by balancing out the contributions ($w_{\text{1}}=\text{0}.\text{4}$ with SBERT and $w_{\text{3}}=\text{0}.\text{4}$ with DistilBERT) to leverage semantic congruency and scaleability. As an example, the semantic similarity in SBERT ensures that the term pairs are intelligible (e.g., in religion clusters, there are terms such as prayer and faith), whereas the efficiency of DistilBERT handles the size of the dataset (e.g., in religion clusters). The ablation experiments demonstrate that dynamic weighting prevents the negative results of over-reliance on the size of RoBERTa (e.g., over-reliance on magnitude) and that clusters are driven by domain-specific intent rather than architecture biases.

**Table 3:** Clustering performance comparisons on 20 Newsgroups.

Model	Evaluation metrics			
	Silhouette	ARI	NMI	UMass
SBERT	0.41	0.48	0.45	0.50
RoBERT	0.47	0.54	0.51	0.56
DistilBERT	0.39	0.44	0.41	0.46
Static ensemble	0.43	0.49	0.46	0.51
GA-Driven ensemble	0.59	0.65	0.62	0.70

Table 4 on Stack Overflow, where there are no ground-truth labels, the improvements of the GA-driven ensemble in Silhouette Score and Topic Coherence are + 14% and +17% over the figures of the static fusion, respectively. RoBERTa (Silhouette = 0.43) and SBERT (Topic Coherence = 0.42) demonstrate wise work but can unlock technical terminology (e.g., “React hooks” vs. Redux architecture) only to some extent. The GA-based approach addresses this problem through dynamic weight change ($w_{\text{2}}=\text{0}.\text{5}$ in RoBERTa, $w_{\text{1}}=\text{0}.\text{4}$ in SBERT) to prefer precision in context in technical areas. As an example, evolved weights ensure clusters keep apart expressions like state management (React) and Redux actions, which are mixed with other expressions due to inflexible fusion. Topic Coherence improvements highlight the fact that GA can optimize the co-occurrence of terms (e.g., useEffect and useState in React cluster) and thus transform raw embeddings into domain-specific categories. These results approve the usefulness of the framework in high-variability contexts that are unlabeled and where geometric isolation and semantic uniformity are critical to actionable information.

**Table 4:** Clustering performance comparisons on Stack Overflow.

Model	Evaluation Metrics	
	Silhouette	UMass
SBERT	0.37	0.42
RoBERT	0.43	0.49
DistilBERT	0.40	0.42
Static ensemble	0.41	0.43
GA-Driven ensemble	0.55	0.60

## 5. Discussion

**Fig 2:**
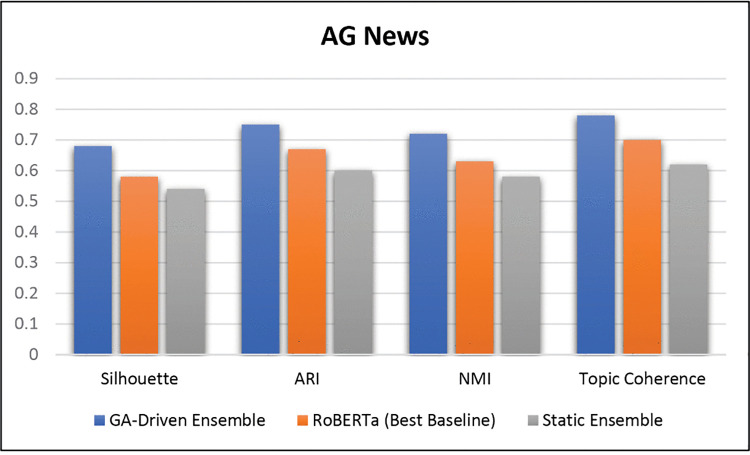
Clustering performance comparison on AG News.

**Fig 3:**
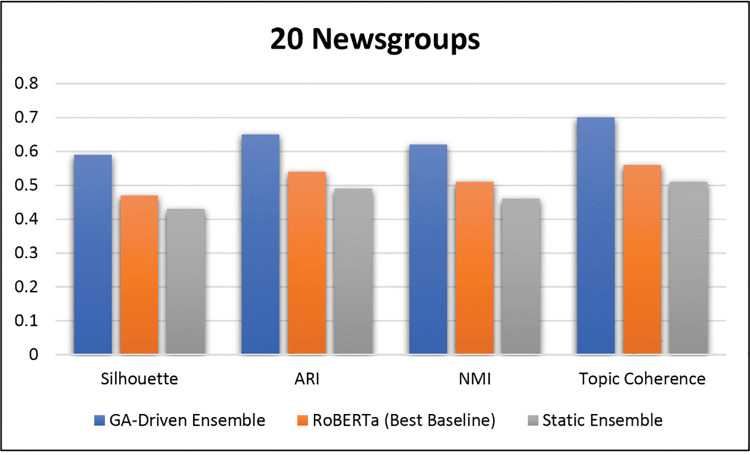
Clustering performance comparison on 20 Newsgroups.

**Fig 4:**
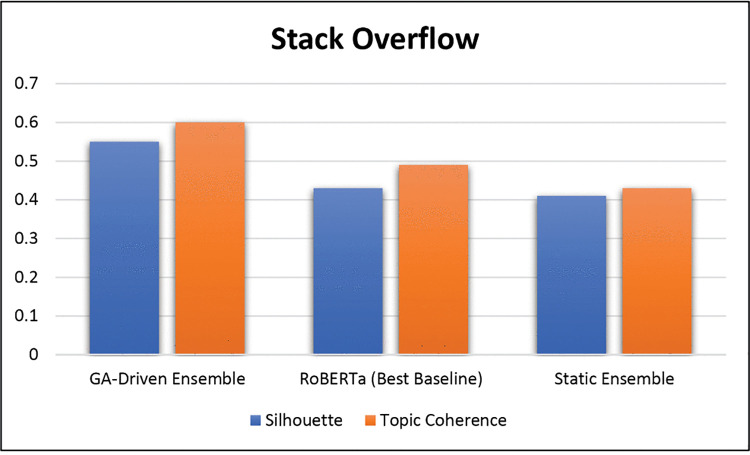
Clustering performance comparison on Stack Overflow.

As we can observe in the summary of the experimental results presented in Figs 2, 3, and 4, and Tables 2, 3 and 4, proposed GA-driven ensemble performs better than standalone models and standalone fusion across all data sets. The following is a breakdown of the reasons behind this, to tie the observed performance to the developed weight distributions and the characteristics of each dataset.

AG News Formal Polysemic Headlines. The GA weight the most to RoBERTa ${\;w}_{\text{2}}=\text{0}.\text{6}$, intermediate to SBERT${\;w}_{\text{1}}=\text{0}.\text{3}$, and least to DistilBERT $w_{\text{3}}=\text{0}.\text{1}$, as illustrated in Fig 2. This distribution indicates the better disambiguation of polysemous words by RoBERTa due to its ability to dynamically mask and train longer, which increases the focus on the modifier phrases. Considering the example of distinguishing between what is known as Apple stock price (Business) and Apple carbon footprint (Science) is based on the identification of contextual cues that RoBERTa is more successful at capturing compared to SBERT. Nevertheless, it can occasionally be the case that RoBERTa misclusters very short headlines that are not richly contextualized (e.g., the release of a new iPhone by Apple), and the global semantic similarity of SBERT offers a stabilizing influence. The uniform weights ensemble (static) dilutes the accuracy of RoBERTa, which results in a reduced ARI and coherence (see Table 2). The GA therefore effectively augments the strengths of RoBERTa whilst keeping the safety net of SBERT and cannot be achieved in fixed weight schemes.

20 Newsgroups, overlapping topics. Fig 3 shows the balanced distribution of the GA between SBERT $w_{\text{1}}=\text{0}.\text{4},$ and DistilBERT $w_{\text{3}}=\text{0}.\text{4},$ with $w_{\text{2}}=\text{0}.\text{2},$ RobERTa being the sole recipient of the allocation. This trend is because deep contextualization of RoBERTa is more prone to overfitting the Usenet posts with their noisy and colloquial language in clusters. The contrastive training model of SBERT generates a homogeneous space of embedding, where similar words in terms of semantics, such as the notions of prayer and faith, are clustered even in the non-scholarly text, whereas DistilBERT provides scalability to the 18,846 documents in the dataset. The GA consequently inhibits the contribution of RoBERTa since it does not want to be fragmented and uses the complementary benefits of SBERT (cohesion) and DistilBERT (efficiency). The non-dynamic unified weighting conditions the RoBERTa to add coherence with an equal probability, which lowers the coherence (Table 3). The outcome of this experiment shows that the GA can down weight models which do not have a good fit in a domain.

Stack Overflow: Technical Jargon. The GA values RoBERTa $w_{\text{2}}=\text{0}.\text{5},$ and SBERT $w_{\text{1}}=\text{0}.\text{4},$ with little contribution $w_{\text{3}}=\text{0}.\text{1},$ by DistilBERT (Fig 4). RoBERTa is also good at capturing complex discourse patterns of technical forums, i.e., the difference between closely similar words such as useEffect (React) and dispatch (Redux). The semantic similarity of SBERT can be used to cluster conceptually similar but lexically dissimilar words (e.g., didMount and useEffect) so that they are not disintegrated. The fact that DistilBERT has lower weight indicates that it has less contextual fidelity to subtle technical differences. By making all the contributions equal, the static ensemble degrades the accuracy required to make correct grouping (Table 4). The weights developed by the GA therefore attain a synergy which trades off context-depth and semantic stability, which is important in unlabeled technical data.

In all the datasets, the quality of weights dynamically adjusted by the GA according to the indicators of the quality of clustering (Silhouette, ARI, Coherence) is critical. The results of the analysis prove that the evolved weight distributions are explainable and correspond to the strengths of each embedding model that have been known, which confirms the framework as adaptable and offers to act on the similar unsupervised tasks.

## 6. Conclusion

The study introduces a GA-based ensemble model which provides a novel definition to unsupervised text clustering as it addresses fundamental weaknesses of fixed fusion models and independent contextual embedding models. The framework bridges the gap between inflexible, uniform averaging and domain-aware optimization by dynamically changing the weights of SBERT, RoBERTa, and DistilBERT using GAs. Critical innovations involve L2-normalization and a sum-to-one constraint to ensure equitable integration of models, without models with inherently larger embedding magnitudes (e.g., RoBERTa in AG News) dominating and sum-to-one constraint ensuring interpretable weight values (e.g., $w_{\text{2}}=\text{0}.\text{6}$ is contextual precision of RoBERTa). Unlike fixed ensembles, which undermine model performance (e.g., by uniformly averaging in Stack Overflow), the GA-driven method progressively updates weights depending on dataset-specific tasks: giving preference to contextual complexity of RoBERTa with technical jargon in Stack Overflow, semantic similarity of SBERT with similar topics in 20 Newsgroups, and performance of DistilBERT with large-scale AG News cl Such dynamic fusion ensures that clusters may adhere to domain intent (e.g., decoupling of react hooks and Redux architecture) and retain computational tractability.

The cross-domain generalizability and strength of the framework to high-dimensional spaces are supported by a systematic ablation and case studies. The mechanisms of tournament selection, Gaussian mutation and elitism ensure that the diversity of the population remains, without premature amalgamation in complex fitness scenarios (such as vague polysemous terms like Apple in AG News). In the case of labeled datasets (AG News), ARI and NMI indicate an increase of +10%−15% over baselines, whereas unlabeled datasets (StackOverflow) achieve 0.60 Topic Coherence, which is 0.17 higher than static fusion. These results highlight the capacity of the GA to supplement accuracy, efficiency, and domain-specific compromises but not relying on ground-truth labels. The flexibility of the framework to linguistic diversity, technicality, and scale is supported by cross-dataset substantiation of AG News (formal headlines), 20 Newsgroups (casual text), and Stack Overflow (technical jargon). As an example, SBERT and DistilBERT jointly deal with casual vagueness in 20 Newsgroups ($w_{\text{2}}$=0.4, $w_{\text{3}}$=0.4) whereas RoBERTa performs best in technical ones ($w_{\text{2}}$=0.5) to disentangle complex patterns of discourse.

This paper advances unsupervised clustering of heuristic pipelines to adaptive, label-free optimization by systematically outperforming the static ensembles and standalone models on heterogeneous datasets. The modular construction of the GA-driven framework makes it easier to combine the emerging models (e.g., BERT-wwm, ELECTRA) and the hybrid goals (e.g., Pareto front analysis in multi-objective optimization). Real-life uses include NLP in daily applications, such as real-time question routing over technical fora, or cross-domain news classification. Future research can generalize the framework to semi-supervised setting and explore distributed GA optimization on huge datasets. This work reduces the gap between evolutionary computation and deep contextual learning by transforming raw embeddings into actionable groupings with no labeled data, and sets a new benchmark of a solid and understandable clustering in unsupervised environments.

## References

[1] Feldman R, Sanger J. The text mining handbook: advanced approaches in analyzing unstructured data. Cambridge University Press. 2007. doi: 10.1017/CBO9780511546914

[2] Stack Overflow Annual Developer Survey. Available from: https://survey.stackoverflow.co/2023/#overview

[3] Mikolov T, Chen K, Corrado G, Dean J. Efficient estimation of word representations in vector space. arXiv preprint arXiv:1301.3781. 2013.doi: 10.48550/arXiv.1301.3781

[4] Pennington J, Socher R, Manning CD. Glove: Global vectors for word representation. In: Proceedings of the 2014 conference on empirical methods in natural language processing (EMNLP). 2014. pp. 1532–43. doi: 10.3115/v1/D14-1162

[5] Levy O, Goldberg Y. Linguistic regularities in sparse and explicit word representations. In: Proceedings of the eighteenth conference on computational natural language learning. 2014. pp. 171–180. doi: 10.3115/v1/W14-1618

[6] Devlin J, Chang MW, Lee K, Toutanova K. Bert: Pre-training of deep bidirectional transformers for language understanding. In: Proceedings of the 2019 conference of the North American chapter of the association for computational linguistics: human language technologies, volume 1 (long and short papers). 2019. pp. 4171–86. doi: 10.18653/v1/N19-1423

[7] Liu Y, Ott M, Goyal N, Du J, Joshi M, Chen D, et al. Roberta: A robustly optimized bert pretraining approach. arXiv preprint arXiv:1907.11692. 2019. doi: 10.48550/arXiv.1907.11692

[8] Raiaan MA, Mukta MS, Fatema K, Fahad NM, Sakib S, Mim MM, et al. A review on large language models: Architectures, applications, taxonomies, open issues and challenges. IEEE Access. 2024;12:26839–74. doi: 10.1109/ACCESS.2024.3365742

[9] Husain H, Wu HH, Gazit T, Allamanis M, Brockschmidt M. Codesearchnet challenge: Evaluating the state of semantic code search. arXiv preprint arXiv:1909.09436. 2019. doi: 10.48550/arXiv.1909.09436

[10] Vakili YZ, Fallah A, Sajedi H. Distilled BERT model in natural language processing. In: 2024 14th International Conference on Computer and Knowledge Engineering (ICCKE). IEEE; 2024. pp. 243–50. doi: 10.1109/ICCKE65377.2024.10874673

[11] Dietterich TG. Ensemble learning. The handbook of brain theory and neural networks. 2002. pp. 110–25.

[12] Azzouz R, Bechikh S, Ben Said L. Dynamic multi-objective optimization using evolutionary algorithms: a survey. Recent advances in evolutionary multi-objective optimization. Cham: Springer International Publishing; 2016. pp. 31–70. doi: 10.1007/978-3-319-42978-6_2

[13] Shahapure KR, Nicholas C. Cluster quality analysis using silhouette score. In: 2020 IEEE 7th international conference on data science and advanced analytics (DSAA). IEEE; 2020. pp. 747–8. doi: 10.1109/DSAA49011.2020.00096

[14] Reimers N, Gurevych I. Sentence-bert: Sentence embeddings using siamese bert-networks. arXiv preprint arXiv:1908.10084. 2019. doi: 10.48550/arXiv.1908.10084

[15] Ortakci Y. Revolutionary text clustering: investigating transfer learning capacity of SBERT models through pooling techniques. Eng Sci Technol: Int J. 2024;55:101730. doi: 10.1016/j.jestch.2024.101730

[16] Ludwig DW 2nd, Guptil C, Alexander NR, Zhalnina K, Wipf EM-L, Khasanova A, et al. SetBERT: the deep learning platform for contextualized embeddings and explainable predictions from high-throughput sequencing. Bioinformatics. 2025;41(7):btaf370. doi: 10.1093/bioinformatics/btaf370 40563247 PMC12245400

[17] Jain AK. Data clustering: 50 years beyond K-means. Pattern Recogn Lett. 2010;31(8):651–66. doi: 10.1016/j.patrec.2009.09.011

[18] Zeng F, Li J, Tang X, Yang Y. A Survey of Unsupervised Clustering Methods Driven by Information Completeness: Challenges, Advances, and Future Directions. In: Proceedings of the 2024 2nd International Conference on Computer, Internet of Things and Smart City. 2024. pp. 153–9. doi: 10.1145/3731867.3731893

[19] Ram A, Jalal S, Jalal AS, Kumar M. A density based algorithm for discovering density varied clusters in large spatial databases. Int J Comput Appl. 2010;3(6):1–4. doi: 10.5120/739-1038

[20] Wolpert DH. Stacked generalization. Neural Netw. 1992;5(2):241–59. doi: 10.1016/S0893-6080(05)80023-1

[21] Haque MN, Noman N, Berretta R, Moscato P. Heterogeneous ensemble combination search using genetic algorithm for class imbalanced data classification. PLoS One. 2016;11(1):e0146116. doi: 10.1371/journal.pone.0146116 26764911 PMC4713117

[22] Sabah A, Tiun S, Sani NS, Ayob M, Taha AY. Enhancing web search result clustering model based on multiview multirepresentation consensus cluster ensemble (mmcc) approach. PLoS One. 2021;16(1):e0245264. doi: 10.1371/journal.pone.0245264 PMC7810326 33449949

[23] Zhou ZH, Feng J. Deep forest. Natl Sci Rev. 2019;6(1):74–86. doi: 10.1093/nsr/nwy108 34691833 PMC8291612

[24] Ghezelbash R, Maghsoudi A, Shamekhi M, Pradhan B, Daviran M. Genetic algorithm to optimize the SVM and K-means algorithms for mapping of mineral prospectivity. Neural Comput Appl. 2023;35(1):719–33. doi: 10.1007/s00521-022-07766-5

[25] Whitley D. A genetic algorithm tutorial statistics and computing. 4. doi: 10.1007/BF00175354

[26] Liu J, Zhang B, Mei Q, Li X, Zou Y, Jiang Z, et al. Dcevo: Discriminative cross-dimensional evolutionary learning for infrared and visible image fusion. In: Proceedings of the IEEE/CVF Conference on Computer Vision and Pattern Recognition. 2025. pp. 2226–35. doi: 10.1109/CVPR52734.2025.00213

[27] Rane N, Choudhary SP, Rane J. Ensemble deep learning and machine learning: applications, opportunities, challenges, and future directions. Stud Med Health Sci. 2024;1(2):18–41. doi: 10.48185/smhs.v1i2.1225

[28] Siranjeevi H, Venkatraman S, Raja SP. EBPGA: extractive text summarization using binary particle swarm optimization and masked genetic algorithm. IEEE Transac Comput Soc Syst. 2025. doi: 10.1109/TCSS.2025.3583893

[29] Huang JY, Tung CL, Lin WZ. Using social network sentiment analysis and genetic algorithm to improve the stock prediction accuracy of the deep learning-based approach. Int J Comput Intell Syst. 2023;16(1):93. doi: 10.1007/s44196-023-00276-9

[30] Chitty-Venkata KT, Emani M, Vishwanath V, Somani AK. Neural architecture search for transformers: a survey. IEEE Access. 2022;10:108374–412. doi: 10.1109/ACCESS.2022.3212767

[31] Shanthi DL, Chethan N. Genetic algorithm based hyper-parameter tuning to improve the performance of machine learning models. SN Comput Sci. 2022;4(2):119. doi: 10.1007/s42979-022-01537-8

[32] Xu J, Xu B, Wang P, Zheng S, Tian G, Zhao J, et al. Self-taught convolutional neural networks for short text clustering. Neural Netw. 2017;88:22–31. doi: 10.1016/j.neunet.2016.12.008 28157556

[33] Zarenia E, Far AA, Rezaee K. Automated multi-class MRI brain tumor classification and segmentation using deformable attention and saliency mapping. Sci Rep. 2025;15(1):8114. doi: 10.1038/s41598-025-92776-1 40057634 PMC11890586

[34] Liu Q, Kusner MJ, Blunsom P. A survey on contextual embeddings. arXiv preprint arXiv:2003.07278. 2020. doi: 10.48550/arXiv.2003.07278

[35] Huang J, Tang D, Zhong W, Lu S, Shou L, Gong M, Jiang D, Duan N. WhiteningBERT: An easy unsupervised sentence embedding approach. In: Findings of the association for computational linguistics: EMNLP 2021. 2021. pp. 238–44. doi: 10.18653/v1/2021.findings-emnlp.23

[36] Li J, Wei Y, Zhang W, Wang C. ESDA: Zero-shot semantic segmentation based on an embedding semantic space distribution adjustment strategy. Image Vis Comput. 2025;155:105456. doi: 10.1016/j.imavis.2025.105456

[37] Tao W, Su X, Wan J, Wei H, Zheng W. Vulnerability detection through cross-modal feature enhancement and fusion. Comput Security. 2023;132:103341.

[38] He Q, Wang K. A hotspot-aware personalized news recommendation mechanism based on DistilBERT-TC-MA. Int J Distribut Syst Technol. 2024;15(1):1–9. doi: 10.4018/IJDST.339565

[39] Miles S, Yao L, Meng W, Black CM, Miled ZB. Comparing PSO-based clustering over contextual vector embeddings to modern topic modeling. Inform Process Manage. 2022;59(3):102921. doi: 10.1016/j.ipm.2022.102921

[40] Abualigah LM, Khader AT, Al-Betar MA. Unsupervised feature selection technique based on genetic algorithm for improving the text clustering. In: 2016 7th international conference on computer science and information technology (CSIT). IEEE; 2016. pp. 1–6. doi: 10.1109/CSIT.2016.7549453

[41] Bezdek JC, Boggavarapu S, Hall LO, Bensaid A. Genetic algorithm guided clustering. InProceedings of the First IEEE Conference on Evolutionary Computation. IEEE World Congress on Computational Intelligence. IEEE; 1994. pp. 34–39. doi: 10.1109/ICEC.1994.350046

[42] Stevens K, Kegelmeyer P, Andrzejewski D, Buttler D. Exploring topic coherence over many models and many topics. In: Proceedings of the 2012 joint conference on empirical methods in natural language processing and computational natural language learning. 2012. pp. 952–61. https://dl.acm.org/doi/10.5555/2390948.2391052

[43] Newman D, Lau JH, Grieser K, Baldwin T. Automatic evaluation of topic coherence. In: Human language technologies: The 2010 annual conference of the North American chapter of the association for computational linguistics. 2010. pp. 100–8. https://dl.acm.org/doi/10.5555/1857999.1858011

[44] Linda S, Minz S, Bharadwaj KK. Effective context-aware recommendations based on context weighting using genetic algorithm and alleviating data sparsity. Appl Artif Intell. 2020;34(10):730–53. doi: 10.1080/08839514.2020.1775011

[45] Song W, Qiao Y, Park SC, Qian X. A hybrid evolutionary computation approach with its application for optimizing text document clustering. Exp Syst Appl. 2015;42(5):2517–24. doi: 10.1016/j.eswa.2014.11.003

[46] Zhang X, Zhao J, LeCun Y. Character-level convolutional networks for text classification. Advances in neural information processing systems. 2015. pp. 28. PMC4831869 27087766

[47] Lang K. Newsweeder: Learning to filter netnews. InMachine learning proceedings. Morgan Kaufmann; 1995. pp. 331–9. doi: 10.1016/B978-1-55860-377-6.50048-7

